# How does the Untire app alleviate cancer‐related fatigue? A longitudinal mediation analysis

**DOI:** 10.1002/pon.5886

**Published:** 2022-01-27

**Authors:** Simon S. Spahrkäs, Anne Looijmans, Robbert Sanderman, Mariët Hagedoorn

**Affiliations:** ^1^ Department of Health Psychology University Medical Center Groningen University of Groningen Groningen The Netherlands; ^2^ Department of Psychology, Health and Technology University of Twente Enschede The Netherlands

**Keywords:** fatigue, longitudinal mediation, mHealth, mindfulness, physical activity, randomized controlled trial

## Abstract

**Objective:**

A waiting‐list randomized controlled trial supported the effectiveness of the multimodal Untire app in reducing cancer‐related fatigue (CRF) in cancer patients and survivors. However, little is known about the causal mechanisms of different app components through which the intervention effect was achieved. We aim to examine whether specifically targeted factors (i.e., fatigue catastrophizing, depression, mindfulness, sleep, and physical activity) mediated the intervention effects of the Untire app on fatigue outcomes.

**Methods:**

Seven hundred ninety‐nine persons with CRF were randomized (2:1) into intervention (*n* = 519) and waiting‐list control (*n* = 280) groups. Self‐report data on the primary outcome fatigue severity and interference and the abovementioned potential mediators were collected at baseline and 12 weeks. Participants who completed the 12‐week assessment were included in the analyses (intervention = 159; control = 176). We performed longitudinal multi‐categorical multiple mediation analysis using PROCESS macro to examine whether the potential mediators explained the overall intervention effects.

**Results:**

Improvements in fatigue catastrophizing (bootstrap 95% CI (−0.110; −0.011)), depression (bootstrap 95% CI (−0.082; −0.004)), and mindfulness (bootstrap 95% CI (−0.082; −0.002)), significantly mediated the intervention effect on fatigue severity, whereas sleep quality (bootstrap 95% CI (−0.081; 0.009)), sleep disturbance (bootstrap 95% CI (−0.038; 0.029)), and physical activity (bootstrap 95% CI (−0.068; 0.000)) did not. Similar associations were found for fatigue interference.

**Conclusions:**

Untire app access reduces fatigue severity and interference mainly by decreasing fatigue catastrophizing, depression, and by increasing mindfulness. Supporting these psychological mechanisms is crucial for reducing fatigue among cancer patients and survivors.

## 1 BACKGROUND

Since cancer‐related fatigue (CRF) is the number one side effect of cancer and cancer treatment,^1^ much research has been targeted at determining effective interventions to ameliorate CRF symptoms.^2^ In a recent waiting‐list randomized controlled trial (RCT),^3^ we found evidence for the effectiveness of the Untire self‐management app in reducing CRF in cancer patients and survivors. Cancer‐related fatigue is a profound, complex, multifactorial problem, with many etiologies involving physiological, biochemical, and psychological systems.^4^ Therefore, the Untire app's content has a multimodal design, combining as a self‐management toolbox various components hypothesized to attenuate fatigue. However, little is known about the causal mechanisms of these different app components through which the intervention effect was achieved.

Effective CRF interventions include physical exercise and targeted psychological and mind‐body treatments, whereas pharmacological interventions could not be advised due to limited effectiveness and side effects.^5^ Evidence‐based CRF interventions can address fatigue reduction through different mechanisms.^4^ Exercise interventions aiming to reduce CRF in cancer patients assume that a lack of physical activity and physical breakdown during cancer (treatment) can worsen fatigue.^6^ It is presumed that with exercise interventions, physical activity, strength, and fitness can be increased,^7^ resulting in a reduction in CRF. Reduced CRF, in turn, allows patients to become more capable of carrying out the activities of daily living and thereby helping further improve the overall quality of life (QoL).^8^ Cognitive‐behavioral therapy (CBT) aimed at reducing CRF is based on the assumption that several fatigue‐related cognitions (e.g., catastrophizing thoughts, depression^2^) and behaviors (e.g., difficulties with goal setting, setting boundaries, poor sleep hygiene) are related to the persistence of fatigue.^9^ Targeting emotions, behaviors, and cognitive processes with CBT is assumed to result in less dysfunctional thoughts about fatigue, contributing to fatigue reduction.^10^ Mindfulness‐based cognitive therapy (MBCT) can reduce CRF by fostering deep relaxation and sleep quality via breathing exercises, autogenic training (i.e., teaching the body to respond to own verbal commands fostering relaxation), as well as body scans (i.e., paying attention to parts of the body and bodily sensations in a gradual sequence from head to toes).^11^, ^12^ Despite the variety in research on all kinds of CRF interventions, only about 13% percent of studies to date investigated multimodal CRF interventions.^2^

Although many potential mechanisms of CRF interventions can be assumed, to our knowledge, only a few studies have evaluated mechanisms that mediate treatment effects of CRF interventions. One RCT performing mediation analysis demonstrated that a physical activity intervention with yoga exercises could reduce cancer fatigue via reduced sleep disturbance.^13^ A recent study on pooled data from three RCTs that tested the efficacy of CBT on CRF suggested causal mediation via increased (cognitive) self‐efficacy and decreased fatigue catastrophizing, focusing on symptoms, perceived problems with activity, and depressive symptoms.^10^ Another RCT on CBT showed that cancer patients experienced a significant increase in fatigue‐related self‐efficacy, with greater self‐efficacy associated with decreased fatigue severity. Contrary, however, there was no evidence that changes in physical activity, exercise capacity, perceived physical activity, fatigue catastrophizing, or emotional functioning mediated CBT's effect on fatigue.^14^

The Untire self‐management app has a multimodal set‐up that combines most of the abovementioned evidence‐based treatment approaches to reduce cancer patients' and survivors' disabling fatigue.^15^ The self‐management app is based on face‐to‐face therapy for CRF, including energy conservation, activity management, restful sleep, mindfulness‐based stress reduction (MBSR), psychosocial support, CBT, and physical activity exercises,^15^, ^16^ and is in line with the guidelines of the National Comprehensive Cancer Network.^17^

## 2 PURPOSE OF THE RESEARCH

In the Untire app RCT, the primary analyses showed that the multimodal Untire app could effectively reduce fatigue symptoms (i.e., fatigue severity and interference). As a next step, we explore the potential mediating role of psychological factors (i.e., fatigue catastrophizing, depression, mindfulness, sleep quality, sleep disruption) and physical activity in lowering fatigue.

## 3 METHOD

### 3.1 Ethical approval

The Medical Ethical Committee of the University Medical Center Groningen (UMCG), the Netherlands, approved the study procedures. We received either ethical approval or a waiver from authorized institutions in the four English‐speaking countries targeted (i.e., Australia, Canada, United Kingdom, and the United States).^18^ Further details are provided in the protocol^15^ and outcome paper.^3^

### 3.2 Study design and setting

The Untire app study is a large‐scale international waiting‐list RCT that examined the effectiveness of the Untire intervention in improving fatigue and QoL in cancer patients and survivors. The Untire self‐management app was launched worldwide in March 2018, and study enrollment took place until October 2018. We recruited potential participants online via paid social media advertisements on Facebook and Instagram.^19^ We targeted patients and survivors of ≥18 years old who experienced fatigue at moderate/severe levels (i.e., an average composite score of ≥3 on items 1, 2, and 3 of the Fatigue Symptom Inventory [FSI]^20^, ^21^) and owned a smartphone. Exclusion criteria were a diagnosis of and receiving treatment for a severe mental disorder (i.e., major depression, psychotic disorder, anxiety disorder, or addiction) as these persons may need more intensive treatment than offered by the app.^3^ Also excluded were persons diagnosed with chronic fatigue syndrome, myalgic encephalomyelitis, or fibromyalgia as the app focuses on improving physical activity, which is not recommended and could potentially be harmful to these patients.^22^ After having completed the baseline questionnaire, 799 participants were randomized (2:1) into intervention (*n* = 519) and waiting‐list control (*n* = 280) groups. Participants in the intervention group received access to the Untire app at the start of the trial, whereas the control group was treated as usual (TAU). This means that control group participants could participate in other programs to reduce CRF. However, little interference of TAU was expected since the usual treatment options for CRF symptoms are limited and rarely discussed by care professionals.^23^ After 12 weeks, the control participants also received the intervention (i.e., 1 year of free app access). Participants received e‐mail invitations and reminders at 4, 8, 12, and 24 weeks to complete the questionnaires.

### 3.3 Untire app

The Untire app intervention is based on evidence‐based methods for patients with CRF in clinical practice and comprises four modules, these are, *My themes, My exercises, Physical activity,* and *Tips*. It is hypothesized that the app addresses dysfunctional thoughts and sleep hygiene via CBT and psycho‐education (My themes). Further, it is hypothesized that the app reduces stress and improves sleep via MBSR (My exercises), helps to improve physical fitness through exercise instructions and activity management (Physical Activity), and empowers via positive psychology (Tips).^11^, ^12^, ^24^ Additionally, through *Quick scans*, participants get weekly insight into their fatigue levels, burden, happiness, satisfaction, and energy leaks over time. Participants in the intervention group were instructed to use the app at least once a week.

### 3.4 Measurements

#### 3.4.1 Dependent variables

The primary outcome is the change in fatigue severity and fatigue interference from baseline to 12 weeks, assessed with the self‐report questionnaire FSI.^20^ Fatigue severity was assessed by calculating the average of three severity items (Cronbach's *α* = 0.71) and fatigue interference by calculating the average of seven interference items (Cronbach's *α* = 0.91). All items were completed on an 11‐point Likert scale ranging from 0 (not at all fatigued/no interference) to 10 (as fatigued as I could be/extreme interference). A higher score indicates higher fatigue severity or interference.

#### 3.4.2 Potential mediators

The following potential mediating factors were assessed fatigue catastrophizing, depression, mindfulness, sleep quality, sleep disturbance, and physical activity. The 13‐item Fatigue Catastrophizing Scale was adapted from the Pain Catastrophizing Scale,^25^ calculating a sum score ranging from 13 (never catastrophizing about fatigue) to 65 (all of the time fatigue catastrophizing; Cronbach's *α* = 0.93). Depression was measured with the 4‐item depression subscale of the Patient‐Reported Outcomes Measurement Information System (PROMIS‐29),^26^ calculating the average score ranging from 1 (never depressed) to 5 (always depressed; Cronbach's *α* = 0.87). Mindfulness was measured with the 5‐item Mindful Attention Awareness Scale,^27^ calculating the average score ranging from 1 (almost never mindful) to 5 (almost always mindful; Cronbach's *α* = 0.81). Sleep quality was measured with the 1‐item sleep quality subscale of the PROMIS‐29, ranging from 1 (very poor sleep quality) to 5 (very good sleep quality). Sleep disturbance was calculated as the average score on the three‐item sleep disturbance subscale of the Symptom Checklist‐90‐Revised,^28^ ranging from 1 (no sleep disturbance at all) to 5 (extreme sleep disturbance; Cronbach's *α* = 0.66). To measure physical activity*,* we used a self‐constructed item (‘How physically active have you been on average in the past month?’) on an 11‐point Likert‐scale ranging from 0 (not physically active at all) to 10 (extremely physically active).

### 3.5 Statistical analyses

Analyses were conducted in IBM‐SPSS Version 26.0, using *α* = 0.05. We described sample characteristics (i.e., gender, age), treatment outcomes (i.e., fatigue severity and fatigue interference), and potential mediator variables (i.e., fatigue catastrophizing, depression, mindfulness, sleep quality, sleep disturbance, and physical activity) at baseline and 12 weeks, for those who completed the 12‐week assessments (i.e., T12‐completer). To investigate the mediating effects of the potential mediator variables on fatigue outcomes after 12 weeks of Untire app access, we used the PROCESS macro, an observed variable ordinary least squares regression‐based modeling tool.^29^ This method can handle longitudinal mediation analysis, including multiple mediators in a single model. Specifically, we tested a parallel multiple mediator model^29^ that estimates the direct and indirect effects of condition X (intervention vs. control) on Y (fatigue outcomes) through the six proposed mediators measured at 12 weeks for each mediation path while accounting for other mediation paths, as well as for covariates (baseline scores for all mediators and the fatigue outcome). The analysis of covariance model has been supported by prior research comparing different methods for estimating mediated effects.^30^

For the longitudinal mediation analyses, the following steps were taken: (1) we tested intervention effects (i.e., *X* = *intervention* vs. *control*) on potential mediators over 12 weeks (*path‐a*); (2) we tested associations between the potential mediators and fatigue severity and fatigue interference over 12 weeks (*path‐b*); and (3) we tested if associations in potential mediators mediated the relationship between the intervention effect (X) and fatigue outcomes (*path‐ab*). Paths *a* and *b* make up the mediating pathway over 12 weeks, with the mediating effect usually described in the literature as the product of coefficients (*ab*).^31^ The *c′*‐path is the direct (unmediated) 12‐week intervention effect on fatigue outcomes in intervention versus control groups. The multiple mediation analysis provides the mediating effect's coefficients with bias‐corrected bootstrapped confidence intervals (CIs), which is currently an optimal way of performing mediation analysis.^32^ Five thousand bias‐corrected bootstrapped samples and 95% CIs were used in the present analysis. Complete case data were used for this analysis because the bias‐corrected CIs can only be generated with complete data.

## 4 RESULTS

### 4.1 Study sample

In total, 799 participants completed the primary outcome at baseline, of which 335 completed the 12‐week fatigue outcome assessment and were therefore included in the analysis. Table 1 shows that most participants were female (92%), middle‐aged (57.4 ± 9.5 years), and moderate to severely fatigued (severity: 6.5 ± 1.4; interference: 5.7 ± 2.0). Means and standard deviations for the potential mediators are also reported in Table 1. Baseline characteristics (including the potential mediator variables) of the 335 T12‐completers did not differ significantly from the baseline characteristics of the 799 participants who completed the baseline assessment.

**TABLE 1:** Baseline characteristics and 12‐week outcomes of study participants who completed the 12‐week fatigue assessment in the intervention or control group

	Baseline			12 weeks	
	Total	Intervention	Control	Intervention	Control
	(*N* = 335)	(*n* = 159)	(*n* = 176)	(*n* = 159)	(*n* = 176)
Demographics					
Female, N (%)	306 (91.9)	146 (92.4)	160 (91.4)	N/C	N/C
Age, *M* ± SD (years)	57.4 ± 9.5	58.0 ± 10.1	56.9 ± 9.8	N/C	N/C
Primary endpoints					
Fatigue Symptom Inventory (FSI), *M* ± SD					
Severity	6.5 ± 1.4	6.4 ± 1.4	6.6 ± 1.4	5.1 ± 2.1	5.8 ± 1.8
Interference	5.7 ± 2.0	5.6 ± 1.9	5.8 ± 2.0	4.0 ± 2.4	4.8 ± 2.4
Potential mediators, *M* ± SD					
Fatigue Catastrophizing Scale (FCS)	30.3 ± 10.5	30.7 ± 10.9	29.4 ± 10.2	26.6 ± 11.0	29.2 ± 11.0
Depression (PROMIS‐29)	2.5 ± 0.9	2.5 ± 0.9	2.5 ± 0.8	2.2 ± 1.0	2.4 ± 1.0
Mindfulness (MAAS)	3.2 ± 1.0	3.2 ± 1.0	3.2 ± 1.0	3.5 ± 1.0	3.3 ± 1.0
Sleep quality (SLC‐90)	2.5 ± 1.0	2.6 ± 1.0	2.4 ± 1.0	2.8 ± 1.1	2.5 ± 1.0
Sleep disturbance (SLC‐90)	3.0 ± 0.9	2.9 ± 0.9	3.0 ± 0.9	2.8 ± 1.0	3.1 ± 1.0
Physical activity	4.0 ± 2.0	4.0 ± 2.0	3.9 ± 2.0	4.9 ± 2.2	4.3 ± 2.3

*Note*: Fatigue severity (0 = not fatigued at all–10 = as fatigued as I could be); Fatigue interference (0 = no interference–10 = extreme interference); Fatigue catastrophizing (13 = never catastrophizing about fatigue–65 = all of the time catastrophizing about fatigue); Depression (1 = never depressed–5 = always depressed); Mindfulness (1 = almost never mindful–6 = almost always mindful); Sleep quality (1 = very poor sleep quality–5 = very good sleep quality); Sleep disturbance (1 = no sleep disturbance at all–5 = extreme sleep disturbance); Physical activity (0 = not physically active at all–10 = extremely physical active). N/C = no change.

### 4.2 Test of the intervention effect (X) on the mediator over 12 weeks (Δ_*M*_)—a‐path

The self‐management intervention significantly improved outcomes of fatigue catastrophizing, depression, mindfulness, sleep disturbance, and physical activity (Table 2). Sleep quality did not differ significantly for both univariate regression models of fatigue severity and fatigue interference (*p* > 0.05), suggesting that the intervention positively affected all but one potential mediator variable over 12 weeks.

**TABLE 2:** Testing paths a, b, c, and c′

Mediators	Intervention versus control over 12 weeks			
	Fatigue		Fatigue	
	Severity		Interference	
Path a (X on M)	BETA	*p*	BETA	*p*
Fatigue Catastrophizing Scale (FCS)	−0.245	0.003	−0.243	0.003
Depression (PROMIS‐29)	−0.205	0.016	−0.198	0.019
Mindfulness (MAAS)	0.207	0.023	0.193	0.034
Sleep quality (SLC‐90)	0.144	0.149	0.140	0.163
Sleep disturbance (SLC‐90)	−0.203	0.040	−0.194	0.049
Physical activity	0.260	0.013	0.261	0.012
Path b (M on Y)				
Fatigue Catastrophizing Scale (FCS)	0.213	0.005	0.245	<0.001
Depression (PROMIS‐29)	0.173	0.020	0.251	<0.001
Mindfulness (MAAS)	−0.174	0.006	−0.270	<0.001
Sleep quality (SLC‐90)	−0.201	0.004	−0.091	0.129
Sleep disturbance (SLC‐90)	0.023	0.743	0.107	0.083
Physical activity	−0.108	0.044	−0.101	0.029
Path c (total effect)	−0.631	0.002	−0.768	0.001
Path c′ (direct effects)	−0.271	0.126	−0.238	0.209

### 4.3 Test of the effect of the mediator (Δ_*M*_) on the treatment outcome (Δ_*Y*_) over 12 weeks—b‐path

The potential mediators from step 1 were significantly related to fatigue severity (*p* < 0.05, Table 2), except sleep disturbance (*p* > 0.05, Table 2). Sleep quality was significantly associated with a reduction in fatigue severity (*p* < 0.05), although it was not significantly associated with a reduction in fatigue interference (*p* > 0.05).

### 4.4 Test of the mediating effect over 12 weeks—path ab

Testing the mediation effects revealed significant associations for the following mediators: fatigue catastrophizing, depression, mindfulness (*p* < 0.05, Table 3), whereas sleep quality, sleep disturbance, and physical activity did not mediate the intervention effect on fatigue outcomes (*p* > 0.05, Table 3). For instance, the model for fatigue severity shows that fatigue catastrophizing improved significantly on average by 0.24 units in the intervention group compared to the control group after 12 weeks. In turn, the reduction of fatigue catastrophizing contributed to reducing fatigue severity experienced over 12 weeks on average by 0.21 units. The mediation effect corresponds to −0.05 units. Controlling all six tested mediators leaves an unmediated model effect of 0.27 units (c’‐path), whereas the total model effect combined accounts for −0.63 units (c‐path). Standardized coefficients (BETA’s) permitted comparison of effects across different mediators, indicating minor differences between mediators with fatigue catastrophizing having the most influence.

**TABLE 3:** Testing the mediation effect of the six pre‐specified longitudinal‐mediators using the bootstrap approach, ANCOVA‐model

Mediators	Intervention versus control over 12 weeks			
	Fatigue		Fatigue	
	Severity		Interference	
Path ab (indirect effect)	Estimated effect	Bootstrap 95% CI	Estimated effect	Bootstrap 95% CI
Fatigue Catastrophizing Scale (FCS)	−0.052	−0.110; −0.011	−0.060	−0.125; −0.013
Depression (PROMIS‐29)	−0.036	−0.082; −0.004	−0.050	−0.103; −0.007
Mindfulness (MAAS)	−0.036	−0.082; −0.002	−0.052	−0.112; −0.003
Sleep quality (SLC‐90)	−0.029	−0.081; 0.009	−0.013	−0.047; 0.008
Sleep disturbance (SLC‐90)	−0.005	−0.038; 0.029	−0.021	−0.058; 0.006
Physical activity	−0.028	−0.068; <0.001	−0.026	−0.065; 0.003

## 5 DISCUSSION

Our international waiting‐list RCT demonstrated beneficial effects of the Untire app on fatigue outcomes of cancer patients and survivors after 12 weeks of app access. The current mediation analysis provides insights into the key drivers behind the multimodal mHealth intervention. The findings support the hypothesis that changes in fatigue catastrophizing, depression, and mindfulness are underlying mechanisms of the found intervention effects. Minor differences between these mediators were found, with fatigue catastrophizing having the most impact. Our study adds to the limited literature on the role of mediators in (multimodal) CRF management research. Specifically, we could observe that through the Untire app, CRF could be reduced via cognitive processes like fatigue catastrophizing and depressive symptoms. A recent study on CBT for CRF demonstrated similar findings, suggesting that the intervention effect could be achieved via reduced fatigue catastrophizing, focusing on symptoms, perceived problems with activity, and depressive symptoms.^10^ Our study also showed that the Untire app could reduce fatigue by increasing mindfulness levels. This is in line with a systematic review on mindfulness‐based eHealth and mHealth programs for patients with cancer, indicating that these programs can reduce the levels of fatigue, depression, stress, anxiety, sleep problems, and pain, and improve the levels of mindfulness, posttraumatic growth, and some parameters of general health.^33^

Contrary to our hypothesis, we did not identify physical activity and sleep as mediators of the intervention effects on fatigue outcomes, while we had expected this based on evidence‐based guidelines^34^ and earlier studies.^10^, ^12^ Nevertheless, we observed that access to the Untire app was associated with an increase in self‐reported physical activity levels and a reduction in levels of sleep disturbance. Our findings contribute to the mixed findings in the literature on the mediating effect of physical activity on fatigue. Two previous RCTs showed that the effect of CBT promoting physical activity for patients with CRF were not mediated by a change in physical activity or physical fitness,^35^ even when physical activity was measured using actigraphy.^36^ On the contrary, another RCT identified graded exercise therapy (GET) as an essential component of CBT for CRF, resulting in a larger reduction in fatigue than the other components, mediating CRF by an increased level of perceived activity.^37^ However, this does not mean that Get alone would be sufficient to manage severe CRF since it was provided as a component of CBT. Another RCT demonstrated that CBT with GET mediated CRF via self‐efficacy only, but not via physical activity, exercise capacity, perceived physical activity, fatigue catastrophizing, or emotional functioning.^14^ Self‐efficacy is assumed to play a role in fatigue reduction but has unfortunately not been assessed in our study. We also learned that Untire app access could not evoke improvements in sleep quality over 12 weeks, although we learned that sleep quality was still significantly associated with fatigue reduction, suggesting a theoretical base.

A limitation is that we used self‐reported, single‐item questions to assess physical activity and sleep. Future studies could use more extensive questionnaires or objective measures (e.g., wearables), and perhaps such assessments would have painted a different picture. Also, it could be the case that some themes within the app can be further improved (e.g., sleep and physical activities).

### 5.1 Clinical implications

Our research has shown that a multimodal self‐management app can be an effective and advisable toolbox to support fatigued cancer patients and survivors via different mediating pathways. We do not know to what extent each part of the app was used since we offered the Untire app as a ‘black box’ with multiple components. Even though many cancer patients and survivors might prefer a single module within the app, it also seems likely that a combination accumulated to the intervention effect of fatigue reduction. Moreover, we believe that a multimodal intervention has further clinical advantages over single‐component interventions because people can prioritize and choose the components they want to work on.

### 5.2 Study limitations

One advantage is that we simultaneously examined several potential mediators in one model. While we could elucidate which mediators contributed to the intervention effects, unfortunately, we cannot make claims about the dose‐response relationship of engaging in specific in‐app modalities concerning changes in mediators and fatigue outcomes. In addition, our analysis allowed taking baseline and 12‐week assessments into account, meaning that we could gain insights about potential mediators over time but could not zoom into the dynamics of mediators and outcomes within the 12 weeks. It seems plausible that the working mechanism of the self‐management app is not a one‐way causal effect. Another limitation might be the higher dropout rates in the intervention group. Although sample characteristics do not differ significantly between completers and non‐completers of the 12‐week assessment, we know that the sample is relatively selective concerning gender, and type of cancer. Although we do not expect differences between the working mechanisms in men and women, we cannot say for sure because of the few men in the trial. Our sample's high number of women might be due to our Facebook Ads recruitment strategy. We targeted cancer patients and survivors, of which mainly women responded, and we then created look‐alike‐audiences with similar characteristics. Research shows that men are generally less likely to perform health‐protective behaviors than women.^38^ In order to increase the use of such apps among male cancer patients and survivors, recruitment via social media might be adapted. In our study, Facebook Ads with images of male participants were slightly associated with improved uptake among men. A recent study demonstrated that using male‐specific ads can result in a significantly higher proportion of men completing a survey than gender‐neutral ads (38% male vs. 25% gender‐neutral).^39^ In order to stimulate the use of these apps among men, it could also be helpful to tailor information in the app specifically to men, informing users that this information is selected for them.

### 5.3 Future research

This study represents an essential step in mHealth research directed at CRF. More research is required to identify factors and their interplay on the causal pathway of CRF. We now investigated the multimodal intervention as a whole, but future studies could further match the time spent in specific modules to outcomes of these modules. Dose‐response analysis could gain further insights into how interventions work and lead to more effective interventions in the future. Besides, future research could focus on app content personalization, which would support patients' use of the app's most beneficial and personally relevant components.

## 6 CONCLUSIONS

This study showed that access to the multimodal Untire app appears to reduce fatigue severity and interference by reducing fatigue catastrophizing, depression and by increasing mindfulness. Supporting these psychological mechanisms is crucial for supporting patients with fatigue due to cancer and cancer treatment.

## CONFLICT OF INTEREST

None declared. The University Medical Center Groningen received funding from Tired of Cancer BV., the developer of the Untire App, to study its effectiveness independently. Independence is declared in a research agreement.

## Data Availability

The data that support the findings of this study are available from the corresponding author upon reasonable request.
